# The influence of cancer on the reprogramming of lipid metabolism in healthy thyroid tissues of patients with papillary thyroid carcinoma

**DOI:** 10.1007/s12020-024-03993-z

**Published:** 2024-08-15

**Authors:** Agata Zwara, Andrzej Hellmann, Monika Czapiewska, Justyna Korczynska, Alicja Sztendel, Adriana Mika

**Affiliations:** 1 Department of Environmental Analytics, Faculty of Chemistry, University of Gdansk, Gdańsk, Poland; 2 Department of General, Endocrine and Transplant Surgery, Faculty of Medicine, Medical University of Gdansk, Gdańsk, Poland; 3 Department of Pharmaceutical Biochemistry, Medical University of Gdansk, Gdańsk, Poland

**Keywords:** Papillary thyroid cancer, Fatty acids metabolism, Healthy tissue

## Abstract

**Background and objectives:**

Over the years we observed changes in the metabolism of glucose, amino acids, fatty acids (FA) and nucleotides in cancer cells in order to maintain their viability and proliferate. Moreover, as the latest data show, cancer also forces a complete change in the behavior of other tissues. For instance, fat-filled adipocytes are often found in the vicinity of invasive solid human tumors. We investigated the effects of papillary thyroid carcinoma (PTC) on the lipid metabolism of healthy tissue distant from the tumor.

**Method:**

Thyroid tissue was collected from female patients immediately after surgical removal of the entire thyroid gland. Blood samples were collected from PTC patients and healthy volunteers. Real-time PCR assays were performed to analyze the expression of lipogenic genes and a broad panel of FA was determined using the gas chromatography-mass spectrometry method.

**Results:**

The concentration of lipids was higher in paratumor tissue than in healthy thyroid tissue (*p* = 0.005). The lipogenic genes tested were significantly increased in paratumor tissue compared to healthy tissue, especially enzymes related to the synthesis of very long-chain saturated and polyunsaturated FAs (VLCSFAs and PUFAs, respectively) (*p* < 0.001). The FA profile also showed increased levels of C22-C26, VLCSFAs and almost all PUFAs in paratumor tissue (*p* < 0.05).

**Conclusion:**

Our study suggests that a restructuring of lipid metabolism occurs in the adjacent healthy thyroid gland and that the metabolism of VLCSFAs and PUFAs is higher in the paratumor tissue than in the distant tissue of the healthy thyroid gland.

## Introduction

PTC is the most common form of thyroid cancer (80% of all thyroid carcinoma), it originates from follicular cells and can occur at any age. PTC shows an indolent course with a survival rate of 95% over 10 years, as most patients respond positively to surgery and targeted therapy [1]. It is characterized by distinct nuclear features with either papillary or solid growth or invasive features. The papillae are lined by follicular cells that show changes in size and shape, nuclear membrane irregularities (intranuclear furrows and intranuclear pseudo-inclusions) and chromatin features. The classic (conventional) subtype of PTC is the most common histologic subtype of this cancer [2]. Interestingly, the prevalence of PTC is extremely high in developed countries. Moreover, it usually grows slowly and when it spreads, it usually affects adjacent tissue and sometimes metastasizes to regional lymph nodes [3]. The etiology of PTC is complex, with a variety of factors contributing to its development. Among these factors, disorders of lipid metabolism have emerged as a notable area of interest. Lipids, including fatty acids, cholesterol and their derivatives, play a fundamental role in cell structure and function. Tumor cells are metabolically very active and undergo many metabolic reprogramming’s to maintain faster proliferation [4]. Even when sufficient oxygen is available, tumor tissue consumes large amounts of glucose through glycolysis [5]. We have already described changes in amino acids composition in the serum of PTC patients, which serve as a source for protein synthesis and are involved in the biosynthesis of other macromolecules [6]. Lipid metabolism is reprogrammed in tumors, and disruption of blood lipids has been identified as a risk factor for tumorigenesis [7]. Most of the changes are related to the number of polyunsaturated fatty acids (PUFAs) and medium chain fatty acids (MCFAs), the concentrations of which were increased in cancerous tissue compared to healthy adjacent thyroid tissue [4, 8]. FAs fulfil different functions in the cell depending on the group from which they originate. PUFAs are precursors for mediators. As the main component of phospholipids, they also build cell membranes. Monounsaturated fatty acids (MUFAs) are an excellent source of energy for rapidly multiplying cells [9]. MCFAs not only serve as a source of energy, but also regulate glucose and lipid metabolism [10]. Long-chain FAs are not only a source of energy, but are also involved in signal transmission [11]. Recent research suggests that very long chain saturated fatty acids (VLCSFAs) are responsible for fluidity and permeability of cell membranes. Cancer cells stimulate the saturation of their membranes and modulate their biophysical properties by activating de novo lipogenesis. SFAs and MUFAs are less susceptible to lipid peroxidation, therefore this modification may protect them from lipid peroxidation. It also alters membrane dynamics and influences the absorption and efficacy of drugs. Cancer-associated lipogenesis gives cancer cells a significant advantage as it helps them to survive chemotherapeutics but also carcinogens [12].

The study of metabolic pathways using lipidomic methods can serve as a viable method to study cancer metabolism and reveal new mechanisms for the disease. It is also a growing tool for the discovery of new biomarkers for metabolites. However, the size of the PTC tumor can be a major difficulty and often the removal of the entire tumor for histopathological examination is of great importance for a correct diagnosis. Often there is no material left for other, non-routine examinations. Therefore, we have taken healthy thyroid tissues from patients with PTC, but at a different distance from the tumor (para-tumor and healthy tissues), to check how much it influence the lipid metabolism and the composition of the fatty acid profile.

## Methods

### Subjects

Thirty female patients aged 43 ± 13 years who underwent thyroidectomy for lobectomy for PTC at the Thyroid Cancer Centre Medical University of Gdansk from January 1, 2021, to March 31, 2022 were included in the study. Data such as age, sex, preoperative serum autoantibody levels, tumor characteristics, and treatment modalities were obtained from the medical records. Routine laboratory parameters were determined at the Central Clinical Laboratory, at the Medical University of Gdansk, the results of which are collected in Supplementary Table S1. Blood samples were collected from patients before the procedure, it was centrifuged for 20 min at 1500 × *g* and the obtained sera were stored at −80 °C. Thyroid tissues were collected immediately after the surgical removal of the entire thyroid gland. The first fragment (C) was taken from the area close to the tumor, approximately 1 cm away. The second part of the thyroid tissue (F) was obtained from a location distant from the tumor, typically from the contralateral lobe, approximately 5 cm away from the tumor site. Due to small tumor size the tissue samples were collected close to tumor but not directly from the tumor. Otherwise, it could cause misinterpretation by the pathologist. Each sample was frozen in liquid nitrogen immediately after collection and stored in aliquots at −80 °C until analysis. Surgical procedures performed for primary tumors included lobectomy and total thyroidectomy. Standard pathologic diagnoses were based on World Health Organization criteria [13]. Control blood was obtained from a cohort of healthy subjects after exclusion of recognizable pathological thyroid findings by ultrasonography (USG). Specifically, subjects with normal thyroid function test results, including TSH, FT3, FT4, anti-TPO and anti-TG, were selected for blood collection. Serum was immediately frozen at −80 °C to ensure preservation.

### Ethical approval

The study design is in line with the Declaration of Helsinki. Protocol of the study received approval from the Local Bioethics Committee at the Medical University of Gdansk (protocol no. NKBBN/62/2021). Informed consents were obtained from all the patients.

### GC-MS analysis of fatty acids

Serum and tissues percentage share of fatty acids were determined using gas chromatography coupled with mass spectrometry. Briefly, total lipids were extracted from serum samples according to Folch et al. [14]. The extracts were then derivatized into fatty acid methyl esters (FAME), dried under a nitrogen stream and stored at –20 °C until analysis. The FAME were analyzed with a GC-EI-MS QP-2020 NX (Shimadzu, Kyoto, Japan) [15].

### Analysis of mRNA levels by real-time PCR

Total RNA was extracted from frozen tissue samples using the RNeasy Plus Universal Mini Kit (Qiagen, Netherlands) according to the attached protocol and described earlier [16]. Sequences of analyzed genes were described in Supplementary Table S2.

### Statistical analysis

The data analysis was performed in SigmaPlot (Systat Software Inc., San Jose, CA, USA). The Shapiro–Wilk test was used to investigate the normal distribution. Comparisons between two groups (control and PTC patients) were made with the Student’s *t* test. Comparison between two tissues of each patient was made with the paired t-test. All values are presented as mean ± SD. A level of *p* < 0.05 was considered statistically significant.

## Results

### Influence of cancer on fatty acids profile in healthy thyroid tissues

**Fig. 1:**
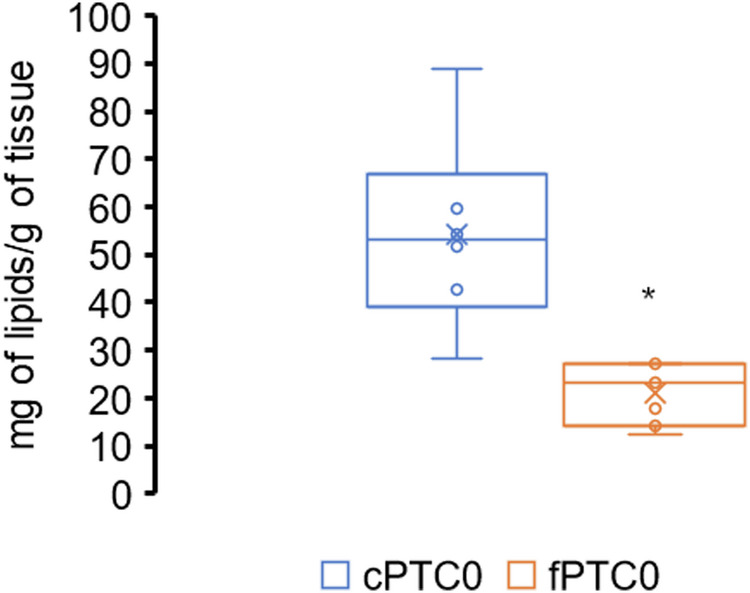
The concentration of lipids in investigated tissues in patients with PTC. cPTC para-tumor tissue, fPTC healthy. Values are mean ± SD. *p* from paired T-test. *p* = 0.005

We investigated the effect of tumor on the fatty acid metabolism in healthy thyroid tissues. For this purpose, we compared healthy tissue sampled near the tumor (para-tumor, cPTC) with healthy tissue sampled as far as possible from the tumor and in the second lobe of the thyroid gland (healthy, fPTC) to evaluate the influence of cancer metabolism on normal cells (Table 1). The most important lipogenic enzymes involved in the synthesis, desaturation and elongation of FAs were increased. The fatty acid translocase gene CD36 and phospholipase A2 (PLA2), which are responsible for the transport of FAs into the cells and the release of arachidonic acid (ARA), among other things, respectively, show higher expression in para-tumor tissue than healthy thyroid (Table 2). Indeed, the concentration of lipids in healthy tissue very close was higher than very far away from the of tumor (Fig. 1).

**Table 1:** Relative levels of mRNA of the enzymes of fatty acid metabolism in tissues of PTC patients measured in two different areas

	cPTC	fPTC	*p*
FASN	0.004 ± 0.001	0.002 ± 0.001	0.002
SCD1	0.029 ± 0.015	0.016 ± 0.007	0.01
ELOVL1	0.021 ± 0.006	0.014 ± 0.004	0.08
ELOVL6	0.003 ± 0.001	0.002 ± 0.001	<0.001
ELOVL2	0.004 ± 0.001	0.002 ± 0.001	<0.001
ELOVL4	0.005 ± 0.003	0.003 ± 0.001	0.02
ELOVL5	0.165 ± 0.053	0.089 ± 0.033	<0.001
FADS1	0.012 ± 0.006	0.005 ± 0.002	<0.001
FADS2	0.037 ± 0.015	0.025 ± 0.014	0.003
PLA2	0.040 ± 0.020	0.017 ± 0.008	0.002
CD36	0.040 ± 0.010	0.020 ± 0.010	<0.001

*CD36* fatty acid translocase, *ELOVLs* fatty acid elongases, *FADS1* fatty acid desaturase 1 (D5D), *FADS2* fatty acid desaturase 2 (D6D), *FASN* fatty acid synthase, *PLA2* phospholipase A2, *SCD1* stearoyl-CoA desaturase-1, *c* close, *f* far of tumor

**Table 2:** Effect of cancer on FAs profile in healthy tissues of thyroid

	Healthy tissue from PTC patients		
	Close to tumor	Far of tumor	*p*
C14	2.22 ± 0.53	2.40 ± 0.74	NS
C16	24.3 ± 3.12	25.5 ± 1.54	NS
C18	11.5 ± 4.36	10.9 ± 3.37	NS
C20	0.28 ± 0.20	0.22 ± 0.10	NS
C22	0.37 ± 0.34	0.11 ± 0.02	0.03
C24	0.43 ± 0.28	0.21 ± 0.11	0.02
C26	0.06 ± 0.02	0.02 ± 0.01	<0.001
C28	0.01 ± 0.01	0.01 ± 0.000	NS
**Total ECFAs**	**39.5** **±** **6.95**	**39.7** **±** **4.63**	**NS**
C15	0.58 ± 0.28	0.46 ± 0.30	NS
C17	0.33 ± 0.11	0.27 ± 0.08	0.05
C19	0.05 ± 0.03	0.04 ± 0.02	NS
C21	0.06 ± 0.06	0.04 ± 0.04	NS
C23	0.12 ± 0.08	0.05 ± 0.02	0.01
C25	0.03 ± 0.02	0.01 ± 0.005	0.02
**Total OCFAs**	**1.19** **±** **0.56**	**0.90** **±** **0.47**	**NS**
**Total SFAs**	**41.3** **±** **7.70**	**41.1** **±** **5.21**	**NS**
C16:1	4.22 ± 0.73	4.33 ± 1.25	NS
C18:1	37.7 ± 6.30	42.9 ± 4.85	0.006
C20:1	0.38 ± 0.11	0.42 ± 0.10	NS
C22:1	0.07 ± 0.04	0.07 ± 0.04	NS
C24:1	0.21 ± 0.14	0.18 ± 0.11	NS
**Total MUFAs**	**42.8** **±** **6.38**	**48.2** **±** **4.44**	**0.008**
HDA	Traces	Traces	NT
LA	10.6 ± 4.73	8.49 ± 2.86	NS
ARA	2.83 ± 0.99	1.15 ± 0.29	0.001
DGLA	0.87 ± 0.30	0.33 ± 0.16	0.001
EDA	0.12 ± 0.05	0.14 ± 0.05	NS
DPAn6	0.05 ± 0.02	0.03 ± 0.01	0.005
AdA	0.29 ± 0.10	0.20 ± 0.07	0.014
**Total n6 PUFAs**	**14.8** **±** **5.27**	**10.2** **±** **2.95**	**0.011**
ALA	0.02 ± 0.01	0.01 ± 0.005	NS
EPA	0.17 ± 0.09	0.07 ± 0.03	<0.001
ETA	0.03 ± 0.02	0.02 ± 0.01	NS
DHA	0.66 ± 0.30	0.30 ± 0.16	0.002
DPAn3	0.33 ± 0.12	0.17 ± 0.08	0.034
**Total n3 PUFAs**	**1.17** **±** **0.46**	**0.53** **±** **0.21**	**0.001**
n6/n3	12.2 ± 3.39	20.7 ± 7.84	0.017

*AdA* adrenic acid, *ALA* α-linolenic acid, *ARA* arachidonic acid, *DGLA* dihomo-γ-linolenic acid, *DHA* docosahexaenoic acid, *DPAn3* docosapentaenoic acid n3, *DPAn6* docosapentaenoic acid n6, *ECFAs* even chain fatty acids, *EDA* eicosadienoic acid, *EPA* eicosapentaenoic acid, *ETA* eicosatetraenoic acid, *HDA* hexadecadienoic acid, *LA* linoleic acid, *OCFAs* odd chain fatty acids, *PUFAs* polyunsaturated fatty acids, *SFAs* saturated fatty acids.

Bold represents main groups of fatty acids

The comparison of the FAs profile from these two areas confirms the results of the mRNA tests. The VLCSFAs were increased in the para-tumor tissue compared to the healthy tissue. The levels of n6 and n3 fatty acids were also more than twice as high in the cPTC tissue as in the fPTC (Table 2). The higher ARA level in cPTC than in fPTC was associated with higher gene expression of desaturase 5 (D5D, FADS1, *p* < 0.001), elongase 5 (ELOVL5, *p* = <0.001), elongase 2 (*p* < 0.001) and desaturase 6 (D6D, FADS2, *p* = 0.003) (Table 1). Consequently, increased metabolic pathways activity was associated with higher intake of energy sources. Lower levels of oleic acid and total MUFAs levels indicated this phenomenon in tumor adjacent tissue compared to healthy tissue (Table 2), although higher gene expression of stearoyl-CoA desaturase-1 (SCD1) was detected in cPTC than in fPTC tissue (*p* = 0.015) (Table 1).

### Influence of cancer on serum patients with PTC

Supplementary Table 1 shows the biochemical parameters determined in the blood of 38 HC and 30 PTC patients. In the serum of the PTC patients, we observed only an upward trend in the blood lipid profile compared to the control, and according to the reference data, the values for LDL-cholesterol and total cholesterol were exceeded. The concentrations of albumin and creatinine were lower in the PTC compared to the HC group.

**Fig. 2:**
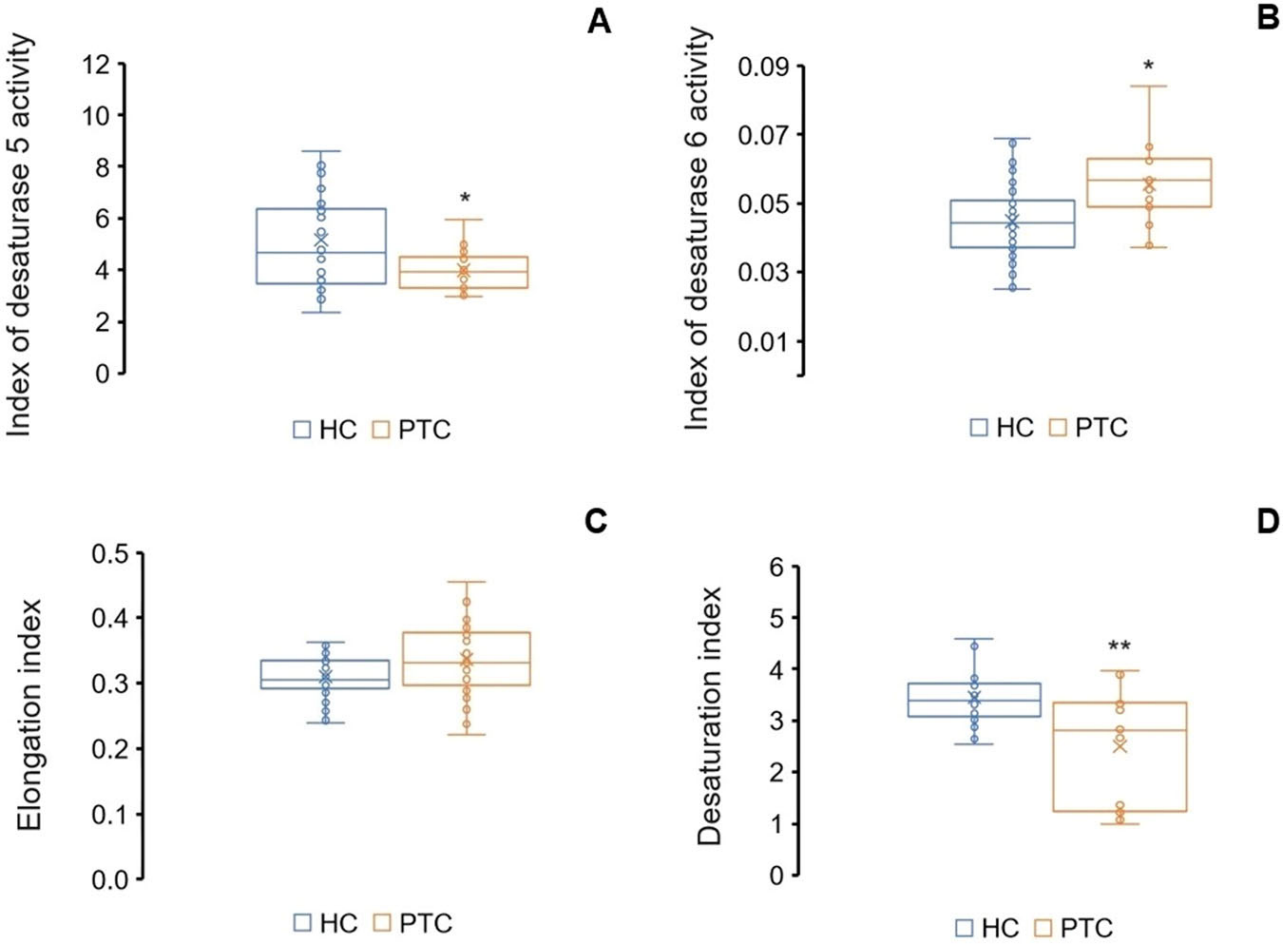
Enzyme activities in the serum of PTC and control. **A**) index of desaturase 5 activity, **B**) index of desaturase 6 activity, **C**) elongation index, **D**) desaturation index. *p < 0.01, **p < 0.001

The comparison of the FAs profile in the serum of PTC and healthy subjects showed many differences. There was a higher level of VLCSFAs as well as almost the entire group of n6 PUFAs (Table 3). Based on the enzyme activities calculated from the FAs serum, we were able to speculate about the activity of the lipogenic enzymes in the missing tumor tissue. Estimates of FAs enzyme activity (EAE) were calculated as the ratio of product-to-substrate for D5D: ARA/ dihomo-γ-linolenic acid (DGLA) and D6D - DGLA/linoleic acid (LA) [17]. The other activities, such as the conversion of oleic acid to stearic acid (desaturation index) and the conversion of steric acid to palmitic acid (elongation index), allowed us to infer the activity of the enzymes SCD1 and ELOVL6 in tumors. The lower D5D activity found in the serum of PTC patients (*p* = 0.003) may indicate that the lack of differences in ARA levels in the sera of the study participants is due to PLA2 activity and not just D5D. In turn, desaturase 6 activity was significantly higher in the serum of PTC patients than in healthy controls (*p* = 0.003) (Fig. 2).

**Table 3:** Profile of fatty acids (%) in the serum of patients with PTC and control

FA	Healthy serum	Cancer serum	*p*
	Control	PTC	
C14	1.20 ± 0.45	0.95 ± 0.28	0.02
C16	22.6 ± 1.66	21.8 ± 1.60	NS
C18	7.33 ± 0.74	7.55 ± 0.86	NS
C20	0.09 ± 0.03	0.13 ± 0.04	<0.001
C22	0.19 ± 0.05	0.25 ± 0.08	0.003
C24	0.18 ± 0.07	0.26 ± 0.08	<0.001
C26	0.011 ± 0.000	0.014 ± 0.01	NS
VLCSFAs (C22 + C24 + C26)	0.37 ± 0.12	0.51 ± 0.17	0.002
**Total ECFAs**	**31.8** ± **1.85**	**31.0** ± **1.48**	**NS**
C15	0.28 ± 0.08	0.30 ± 0.10	NS
C17	0.27 ± 0.06	0.26 ± 0.07	NS
C19	0.019 ± 0.01	0.022 ± 0.01	NS
C21	0.017 ± 0.01	0.023 ± 0.01	0.01
C23	0.059 ± 0.02	0.083 ± 0.02	<0.001
**Total OCFAs**	**0.67** ± **0.15**	**0.69** ± **0.17**	**NS**
**Total SFAs**	**32.80** ± **1.95**	**32.0** ± **1.62**	**NS**
C16:1	3.19 ± 0.70	3.14 ± 0.77	NS
C18:1	25.1 ± 2.34	25.5 ± 2.10	NS
C20:1	0.118 ± 0.05	0.125 ± 0.04	NS
C22:1	0.017 ± 0.01	0.022 ± 0.01	0.04
C24:1	0.28 ± 0.09	0.35 ± 0.15	0.04
**Total MUFAs**	**28.9** ± **2.48**	**29.3** ± **2.17**	**NS**
HDA	0.011 ± 0.002	0.010 ± 0.000	NS
LA	27.7 ± 2.74	27.0 ± 3.00	NS
EDA	0.119 ± 0.04	0.147 ± 0.04	0.006
DGLA	1.22 ± 0.27	1.46 ± 0.31	0.004
ARA	6.19 ± 1.49	6.28 ± 0.88	NS
AdA	0.10 ± 0.03	0.14 ± 0.03	<0.001
DPAn6	0.06 ± 0.02	0.10 ± 0.04	<0.001
**Total n6 PUFAs**	**35.5** ± **2.88**	**34.8** ± **3.04**	**NS**
ALA	0.28 ± 0.14	0.30 ± 0.10	NS
ETA	0.075 ± 0.04	0.078 ± 0.02	NS
EPA	0.76 ± 0.33	0.80 ± 0.33	NS
DPAn3	0.32 ± 0.09	0.41 ± 0.11	0.002
DHA	1.32 ± 0.48	1.49 ± 0.51	NS
**Total n3 PUFAs**	**2.75** ± **0.82**	**3.01** ± **0.79**	**NS**
n6/n3	14.1 ± 4.38	11.86 ± 2.80	0.02

*AdA* adrenic acid, *ALA* α-linolenic acid, *ARA* arachidonic acid, *DGLA* dihomo-γ-linolenic acid, *DHA* docosahexaenoic acid, *DPAn3* docosapentaenoic acid n3, *DPAn6* docosapentaenoic acid n6, *ECFAs* even chain fatty acids, *EDA* eicosadienoic acid, *EPA* eicosapentaenoic acid, *ETA* eicosatetraenoic acid, *HDA* hexadecadienoic acid, *LA* linoleic acid, *OCFAs* odd chain fatty acids, *PUFAs* polyunsaturated fatty acids, *SFAs* saturated fatty acids.

Bold represents main groups of fatty acids

Although the elongation index did not differ in the sera of the investigated groups (*p* = 0.052), SFAs and MUFAs originated from an increased expression of the CD36 gene. Especially since the activity of the SCD1 enzyme was significantly lower in patients with PTC (*p* < 0.001), and lower MCFAs representative, C14, which is substrate of C16, was lower in the serum of patients compared with control (Table 3). The n6/n3 ratio in the serum of PTC patients was lower than in the serum of the control group (*p* = 0.020) (Table 3), similar to that in para-tumor tissue compared to healthy tissue (*p* = 0.017) (Table 2).

**Fig. 3:**
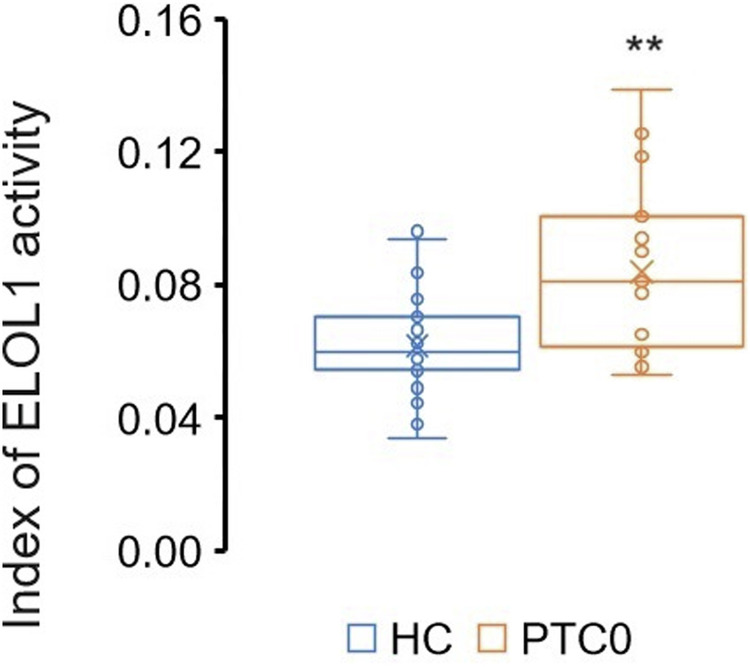
ELOVL1 activity in the serum of PTC and control. *p* < 0.001

Due to the higher level of VLCSFAs in cancer serum compared to control (Table 3) we determined the enzymatic index of ELOVL fatty acid elongase 1 (ELOVL1) in serum, estimated by calculating the product/precursor ratio of the fatty acids involved ((C24 + C22 + C20)/C18). The activity of the ELOVL1 enzyme was significantly higher in patients with PTC compared to control (*p* < 0.001) (Fig. 3). What is interesting, although in para-tumor tissue whole group of VLCSFAs was higher than in healthy tissue (Table 2) we didn’t detect significantly higher gene expression of ELOVL1 in para-tumor tissue compared to healthy tissue (Table 3).

## Discussion

To the best of our knowledge, this is the first study to determine metabolism of FA in normal thyroid tissue from two areas: healthy thyroid tissue adjacent to the cancer (para-tumor tissue) and tissue located as far away from the tumor as possible, from the second lobe of the thyroid gland (healthy tissue). Finally, based on the EAE estimated in the serum of PTC patients, we speculated on the activities of selected enzymes in thyroid tumor. The mechanism underlying the reprogramming of lipid metabolism in PTC remains to be elucidated. In this study, lipidomic analysis showed that fatty acid metabolism differs between para-tumor tissue and healthy tissue.

The first step in this study was to analyze the basic biochemical parameters of the blood of PTC patients and compare their results with the healthy volunteers of study. We observed a significantly lower albumin level in the blood of PTC patients compared to the control, which may be associated with the malnutrition and inflammation that occur during the course of the cancer. The level of this biochemical parameter in the blood can provide information about the progression and prognosis of the disease. Studies show that a low albumin level in the blood is associated with a faster progression of the disease and a higher mortality rate in patients [18]. Blood creatinine levels were also significantly lower in PTC patients compared to the control group, which is also related to malnutrition and can be linked to the patients’ decreasing muscle mass caused by the progression of the cancer [19]. However, despite that possible malnutrition the level of lipid in serum was comparable with control. Also, less than 45% of PTC patients had correct weight of body (Supplementary Table S1). The prevalence of overweight and obesity was increased in PTC cancer, suggesting a possible association between these two diseases. Indisputably, low-grade chronic inflammation, oxidative stress, altered cytokine levels and hormonal changes that occur in obese patients are all factors that may contribute to the occurrence and growth of PTC [20].

Higher enzyme activities in the PTC patients, calculated based on serum FA levels, suggest reprogramming of lipid metabolism not only in cancerous tissue but also in adjacent healthy tissue. This phenomenon was confirmed by significantly higher gene expression in para-tumor tissue compared to healthy tissue in our study. We observed higher ELOVL1 activity, the main enzyme responsible for the production of VLCSFAs, which are important components of membranes. Due to their structure, VLCSFAs increase the stiffness of cell membranes and decrease their permeability [21], thereby protecting cancer cells from potential external threats [22, 23]. Although the expression of ELOVL1 was not statistically significant in para-tumor tissue compared to distant tissue, ELOVL4 catalyzes the synthesis of very long-chain fatty acids [24], and expression of this enzyme was also increased in the adjacent thyroid tissues with cancer examined. Recently, the association between ELOVL4 and various tumors such as gastric cancer has been revealed, and the authors advocate further studies to uncover the possible mechanism of ELOVL4 in cancer progression [25]. Furthermore, in vitro and in vivo mouse experiments from 2023 show that the NOTCH-RIPK4-IRF6-ELOVL4 signaling axis acts as a potent tumor suppressor in squamous cell carcinomas [26]. Overexpression of ELOVL2 and ELOVL5 in para-tumor tissue demonstrates the production of ARA, which is necessary for the generation of signaling and inflammatory lipids [23]. Of course, the endogenous synthesis of ARA is controlled by several synthetic enzymes, including also FADS1, FADS2 and FADS3 [27]. Further, increased activity of enzymes involved in the elongation and desaturation of FAs is necessary for tumor formation and growth [28, 29]. In our study, we also observed overexpression both ELOVL 6 and SCD1 in para-tumor tissue as well as elongation and desaturation indices in PTC patients. In recent years, more and more studies have been carried out on polyunsaturated fatty acid desaturases. Similar to our research, most studies described abnormal expression of FADS2 in many cancers, including liver, lung, breast, esophagus, leukemia, melanoma and other malignant tumors, and showed a significant correlation between FADS2 expression and tumor proliferation, cell migration and invasion, angiogenesis, resistance to radiotherapy, histologic grade, metastasis to lymph nodes, clinical stage and prognosis [30]. Our previous studies indicate increased levels of n3 and n6 in various cancers [16, 31, 32], which may be associated with the possibility of faster proliferation of cancer cells and other factors mentioned above. Similarly, FADS1 is upregulated in some cancer and effectively mediates PUFA synthesis [27]. However, in our study we observed lower FADS1 activity in PTC patients compared to control. N6 PUFA tends to accelerate inflammation, cancer cell proliferation and metastasis while n3 typically counteracts these effects [33]. In our study, the concentration of n3 was higher in para-tumor tissue, which may suggest that n3 is removed from the tumor environment to protect it from ferroptosis, especially since mesenchymal cancer cells have a greater susceptibility to ferroptosis compared to their epithelial counterparts [34].

Cancer cells act on other tissues to obtain the material and energy source necessary for their construction. Many neoplastic tissues are capable of biosynthesizing FAs de novo and rely on newly synthesized FAs to meet the high metabolic demands of rapidly proliferating cells [35] but also influence of others cells [36]. There are good reasons to surround cancer with adipocytes. In addition to their effect on the tumor microenvironment, cancer-associated adipocytes also affect the cancer cells themselves, leading to tumor growth, metastasis and treatment resistance [37, 38]. These effects are mediated by the broad spectrum of adipokines, extracellular vesicles, ketone bodies, glutamine and FAs secreted by cancer-associated adipocytes [37]. With an organ as small as the thyroid gland and the probable lack of fat, the cancer may induce the healthy thyroid cells to produce fatty acids, as our study shows. Of course, further investigations and determination of other proteins involved in FAs metabolism in thyroid tissue are needed, as well as a summary of the results obtained with tumor tissue. The results collected in this way will contribute to the clinical use of potential lipid biomarkers in the diagnosis of thyroid cancer.

The limitations of this study certainly include the small group of PTC patients studied, which should be improved in the future. Another limitation could be that EAEs determined based on serum data may nevertheless also be the result of metabolic changes in other organs, not only in the PTC.

## Supplementary information

Supplementary TableS1

Supplementary TableS2
