# Supplementary material for: The influence of cancer on the reprogramming of lipid metabolism in healthy thyroid tissues of patients with papillary thyroid carcinoma
Source: Endocrine. 2024 Aug 15;87(1):273–80. doi: 10.1007/s12020-024-03993-z (PMC11739254; doi:10.1007/s12020-024-03993-z)
Supplement: Supplementary file 1 — Supplementary TableS1 [file 12020_2024_3993_MOESM1_ESM.doc]

Supplementary Table S1. Anthropometric and laboratory parameters analyzed from PTC patients and healthy control (HC)

|  | HC | PTC | p |
| --- | --- | --- | --- |
| Age (years) | 45.5 ± 10.1 | 43 ± 13 | NS |
| BMI (kg/m^2^) | 25.9 ± 7.53 | 26.3 ± 4.55 | NS |
| Triacylglycerols (mg/dL) | 85.2 ± 36.4 | 95.1 ± 39.4 | NS |
| HDL-C (mg/dL) | 56.0 ± 13.5 | 61.2 ± 12.9 | NS |
| LDL-C (mg/dL) | 111 ± 32.2 | 123 ± 40.1 | NS |
| Total cholesterol (mg/dL) | 189 ± 45.1 | 204 ± 42.4 | NS |
| CRP (mg/L) | 0.94 ± 0.58 | 1.41 ± 1.19 | NS |
| Glucose (mg/dL) | 89.3 ± 8.05 | 93.3 ± 26.6 | NS |
| Albumin (g/L) | 57.4 ± 10.7 | 41.8 ± 2.61 | <0.001 |
| Creatinine (mg/dL) | 0.80 ± 0.11 | 0.68 ± 0.11 | 0.004 |

HC-healthy control; PTC- papillary thyroid cancer; BMI - body mass index; hsCRP – high sensitivity C-reactive protein; HDL-C – high density lipoprotein cholesterol; LDL-C – low density lipoprotein cholesterol; NS—not significant.
