# Supplementary material for: The influence of cancer on the reprogramming of lipid metabolism in healthy thyroid tissues of patients with papillary thyroid carcinoma
Source: Endocrine. 2024 Aug 15;87(1):273–80. doi: 10.1007/s12020-024-03993-z (PMC11739254; doi:10.1007/s12020-024-03993-z)
Supplement: Supplementary file 2 — Supplementary TableS2 [file 12020_2024_3993_MOESM2_ESM.doc]

Supplementary Table S2. List of analyzed genes and their sequences

| FASN | F: CGCTCGGCATGGCTATCT  R: CTCGTTGAAGAACGCATCCA |
| --- | --- |
| SCD1 | F: AACAGTGTGTTCGTTGCCACTT  R: GGTAGTTGTGGAAGCCCTC |
| ELOVL1 | F: CTGTGGCACAACCCTACCTT  R: CTGGGAGATGTGCAGTGAGA |
| ELOVL6 | F: CAAAGCACCCGAACTAGGAG  R: TGGTGATACCAGTGCAGGAA |
| ELOVL2 | F: ATGTTTGGACCGCGAGATTCT  R: CCCAGCCATATTGAGAGCAGATA |
| ELOVL4 | F: GAGCCGGGTAGTGTCCTAAAC  R: CACACGCTTATCTGCGATGG |
| ELOVL5 | F: TAACAGGAGTATGGGAAGGCA  R: ACCAGAGGACACGGATAATCTT |
| FADS1 | F: CCAACTGCTTCCGCAAAGAC  R: GCTGGT GGTTGTACGGCATA |
| FADS2 | F: AAGGGTGCCTCTGCCAACT  R: GATTGTAGGGCAGGTATTTCAGC |
| PLA2 | F: CCTGGATGGAGGAGGAGTGA  R: GATTGTAGGGCAGGTATTTCAGC |
| CD36 | F: AAGTCACTGCGACATGATTAATGG  R: GAACTGCAATACCTGGCTTTTCTC |
| Cyclophilin | F: CGTCTCCTTTGAGCTGT  R: TCGAGTTGTCCACAGTCA |
